# Comparison of clinical outcomes of a corneal wavefront- and topography-guided platforms for laser in situ keratomileusis on virgin eyes: an expanded cohort study

**DOI:** 10.1007/s10792-024-03235-1

**Published:** 2024-07-22

**Authors:** Li Li, Lu Xiong, Zheng Wang

**Affiliations:** 1 Chongqing Eye and Vision Care Hospital, Chongqing, China; 2 Aier School of Ophthalmology, Central South University, Fourth Floor, New Century Mansion, 198 Middle Furong Road, Changsha, 410015 China; 3 Department of Refractive Surgery, Guangzhou Aier Eye Hospital, Guangzhou, China; 4 Aier Institute of Refractive Surgery, Aier Eye Hospital Group, Changsha, China

**Keywords:** Laser in situ keratomileusis (LASIK), Higher-order aberrations, Corneal-wavefront-guided-LASIK, Corneal-topography-guided-LASIK

## Abstract

**Purpose:**

To compare the clinical outcomes of myopiacorrected with corneal-wavefront-guided (CWG) laser in situ keratomileusis (LASIK) with AMARIS 1050S (SCHWIND eye-tech-solutions GmbH & Co. KG) and corneal-topography-guided (CTG) LASIK with WaveLight EX500 (Alcon Laboratories, Fort Worth, TX).

**Methods:**

In this prospective, pseudo-randomized expanded cohort study, a total of 266 patients were subjected to binocular LASIK surgery, either with WaveLight EX500 (WaveLight group) or Amaris 1050S (AMARIS group) platforms. Data related to right eyes were selected for analysis. Corneal higher-order aberration (HOA) was selected as the primary endpoint; while visual acuity and refraction were the secondary endpoints. All the endpoints were assessed at 3 months postoperatively.

**Results:**

There were 134 eyes in the AMARIS group and 132 eyes in the WaveLight group. After 3 months of postoperative follow-up, spherical and coma aberrations were significantly lower (*P* < 0.05) in the WaveLight group (spherical aberration: − 0.104 ± 0.199 µm; coma aberration: − 0.117 ± 0.202 µm) in comparison with the AMARIS group (spherical aberrations: 0.254 ± 0.146 µm; coma aberrations: 0.316 ± 0.297 µm). In the AMARIS group, 96.3% of the eyes achieved an uncorrected distance visual acuity (UDVA) of 20/20 while in the WaveLight group, 96.2% of the eyes achieved an UDVA of 20/20. Furthermore, the mean postoperative manifest refraction spherical equivalent (MRSE) was − 0.02 ± 0.28 in the AMARIS group and − 0.05 ± 0.21 in the WaveLight group (*P* = 0.34).

**Conclusions:**

Both WaveLight EX500 and Amaris 1050S LASIK showed excellent refractive and visual outcomes. In addition, the WaveLight group showed minimal spherical and coma aberrations when compared to the AMARIS group.

## Introduction

Among the different therapeutic modalities available for the correction of visual refractive errors, laser in situ keratomileusis (LASIK) is by far the most frequently used technique that provides satisfactory visual outcomes with relatively better safety [1, 2]. LASIK corrects refractive power in myopia by decreasing central corneal curvature, and in the case of hyperopia, it flattens the paracentral area thereby restoring emmetropia [3]. LASIK surgery typically involves creating a hinged corneal flap from the epithelium, Bowman’s membrane, and the superficial part of the corneal stroma followed by ablation of the posterior basement membrane [4].

LASIK procedure corrects lower order aberrations, however, it may create higher order aberrations (HOAs) because of ablation of corneal tissue and cutting of a flap from the anterior layer of the cornea [5]. These aberrations lead to reduced light and contrast sensitivity, causes glare and halos, and various adverse effects on the postoperative visual quality [6]. Among different LASIK strategies, topography-guided LASIK and wavefront-guided (WFG) LASIK are the latest technologies that have relatively better safety with lower incidence of HOAs in comparison to conventional LASIK [7]. The basic objective of both topography-guided and WFG LASIK is to create custom ablation profiles in order to correct existing HOAs while minimizing the postoperative HOAs.

While WFG LASIK considers all the aberrations of the optical system and converts them to clinically useful data (Zernike polynomials), corneal topography-guided (CTG) treatment, on the other hand, considers aberrations present only in the cornea. CTG utilizes images taken at different unique elevation points to create an elevation profile for individual patients which is compared with an optimum elevation profile. The difference in the patients’ elevation profile and the optimum elevation profile provides the individual ablation profile for the LASIK procedure [8]. Whereas, in case of WFG, the preoperative wavefront aberrometry data is used for creating custom ablation profile [9].

Despite of the perceived advantages of the WFG approach, in clinical studies, it is yet to be conclusively proven to be superior than other LASIK approaches [10]. Furthermore, patients’ treatment history, corneal topography, scotopic pupil diameter, available stroma and centration strategy may also affect the postoperative outcomes [11]. This complicates the selection of the optimal LASIK approach for patients with myopia, hyperopia and astigmatism.

Recent studies have reported that CTG technology is better than WFG ablation as it reduces aberrations caused by refractive surgical procedures, decreases preoperative wavefront aberrations, and improve postoperative visual acuity with fewer HOAs [6, 12, 13]. However, there is limited evidence to advocate that visual outcomes of topography guided ablations are superior than WFG ablation or vice versa in regular corneas [14]. Different platforms that can utilize the custom ablation profiles created by corneal topography and wavefront have become available in recent years [8]. Among the available CTG custom ablation treatment (T-CAT) platforms, the ALCON Contoura vision used in conjunction with the WaveLight EX500 Excimer laser system can successfully correct highly aberrated corneas with irregular astigmatism [14]. Furthermore, T-CAT technology has also been reported to improve the postoperative visual acuity of virgin eyes with lower incidence of HOAs [15]. Among the WFG platforms, the Schwind Amaris platform is reported to be both safe and effective [16]. The Schwind Amaris platform improves corneal aberrations by enabling asymmetric cutting which is used globally except in the United States. In addition, the Schwind Amaris platform selectively removes HOA components within the seventh order, and in the “minimize” mode removes preoperative corneal irregularities, sparing the ablation tissue [17]. However, it is important to understand the differences in clinical outcomes associated with these devices in order to maximize their applicability.

Currently, the incidence of post-operative HOAs has become an important criterion for the making of clinical decision. Although visual and refractive outcomes of LASIK involving WaveLight EX500 and Schwind Amaris platforms have been compared with other platforms, there is no conclusive evidence confirming the superiority of one over the other with respect to the incidence of HOAs [15, 18]. In our previous study, we compared the differences in visual, refractive, and level of ablating decentration between WFG LASIK with Schwind Amaris and CTG LASIK with WaveLight Contoura using different centration strategies for the correction of myopia and myopic astigmatism [19]. However, the paucity of clinical evidence urges an analysis of incidence of HOAs irrespective of the centration strategy with Schwind Amaris and WaveLight EX500 on an expanded cohort of patients. Therefore, in the current expanded cohort study we present the clinical outcomes with specific emphasis on HOAs (visual acuity, refraction, corneal aberrations, and contrast sensitivity) after LASIK using the WaveLight EX500 and Schwind Amaris 1050S platforms.

## Methods

### Study design and patients

This prospective, pseudo-randomized, expanded cohort study included 266 patients who were eligible for receiving binocular LASIK surgery to correct myopia or myopic astigmatism between November 2017 and January 2018. The study received approval from the Medical Ethics Committee of Aier Eye Hospital and patients were enrolled in to the study after providing written informed consent.

The inclusion criteria were patients of age 18–40 years with a documented increase in refraction < 0.50 diopter (D) per year over the last 2 years; preoperative corrected distance visual acuity (CDVA) was better than 20/25; manifest refraction spherical equivalent (MRSE) was less than − 10.0 D and a cylindrical error that was less than − 6.0 D were included. Patients were excluded if they had central corneal thickness less than 480 µm and/or if the residual corneal stroma bed was less than 280 µm, keratoconus or history of ophthalmic surgery. Besides, we ensured that there was excellent alignment between the corneal cylinder and manifest cylinder magnitude and axis was less than a 0.5 D difference in magnitude and less than 15° in the axis. The other inclusion and exclusion criteria were as per the previous publication [19].

### Grouping

A pseudo-randomization was done where patients were included in the order of the first visiting date. Patients whose first visit was on odd numbered date were included in the WaveLight group and received WaveLight EX500 (Alcon, TX, USA) CTG LASIK in both eyes. Patients with an even numbered date of first visit were included in the AMARIS group and received Amaris 1050S (SCHWIND Eye-Tech-Solutions, Kleinostheim, Germany) CWG LASIK in both eyes. All patients were followed for at least 3 months postoperatively and only right eye data were selected for analysis.

### Preoperative and postoperative assessments

Each patient had a rigorous ophthalmic examination preoperatively and 3 months postoperatively. The primary visual outcomes, such as uncorrected distance visual acuity (UDVA), MRSE, CDVA, corneal tomography (Pentacam HR, Oculus, Optikgeräte GmbH), contrast sensitivity (Vector Vision), and corneal aberration data, were collected. Corneal aberrations were assessed on a Vario Topolyzer which recorded the Zernike coefficient of aberrations from the central 6 mm corneal zone. Wavefront aberrations were described by Zernike polynomials using the Optical Society of America (OSA) coefficient standards; HOAs assessed were spherical aberration, coma aberration, vertical coma aberration, and horizontal coma aberration.

### Surgical procedure (T-CAT/CWG LASIK)

To rule out the effect of surgeon’s skill, all the surgical procedures were performed by a single surgeon (WZ). All procedures were performed on Wavelet FS200 (Alcon, Ft Worth, TX, USA) to create a corneal flap with a thickness of 110 μm and a diameter of 6.5 mm.

#### WaveLight topography-guided treatment

The T-CAT surgery was performed with the food and drug administration (FDA) standard mode. The optical zone and transition zone diameter were 6.5 mm and 2.5 mm, respectively [14, 15].

#### SCHWIND custom ablation manager

The ORK-CAM mode was used to correct all corneal aberrations using the asymmetric offset. The optical zone diameter was 6.5 mm [20].

### Study endpoints

The primary endpoint was to assess the HOAs after 3 months of postoperative follow-up. The other endpoints were the parameters of visual acuity.

### Statistical analysis

Statistical analysis and graphical plotting were done with SPSS software (version 22.0, International Business Machines Corp). Data were summarized using descriptive statistics. Continuous data were expressed as mean ± standard deviation (SD). Statistical significance was set at P values < 0.05. The normality of the quantitative data was analyzed using Kolmogorov Smirnov test. T-test was used for normally distributed data; and Wilcoxon test was applied for the comparison of continuous variables with non-normal distribution. Linear regression analysis was used to evaluate the relationship between HOAs and corneal morphological parameters. The differences between the preoperative and postoperative parameters between both groups were analyzed using analysis of variance (ANOVA).

## Results

A total of 266 consecutive patients (63.15% female and 36.46% male) were enrolled. Of which, 132 received T-CAT LASIK using the WaveLight EX500 platform (WaveLight group) and 134 received CWG LASIK using the Amaris ORK-CAM platform (AMARIS group), to correct ametropia. There were no significant differences in the preoperative characteristics between the 2 groups in terms of age, refraction, and central corneal thickness (Table 1).

**Table 1:** Preoperative patient profile for Amaris CWG and EX500 T-CAT

Parameter	T-CAT (n = 131)	CWG (n = 134)	^a^*P*
Sex (M/F)	47/84	50/84	0.81
Age (years)	26.2 ± 5.4 (18 ~ 39)	26.5 ± 5.6 (18 ~ 38)	0.63
Sphere (D)	− 4.97 ± 1.68 (− 1 ~ − 8.75)	− 5.10 ± 1.53 (− 1 ~ − 8.75)	0.51
Cylinder (D)	− 0.87 ± 0.65 (0 ~ − 4.5)	− 0.85 ± 0.81 (0 ~ − 5.0)	0.86
MRSE (D)	− 5.40 ± 1.71 (− 1.63 ~ − 9.13)	− 5.53 ± 1.53 (− 1.13 ~ − 8.88)	0.54
CDVA (logMAR)	0.004 ± 0.015 (00.1)	0.004 ± 0.014 (0 ~ 0.1)	0.81
UDVA (logMAR)	1.014 ± 0.232 (0.4–1.7)	1.048 ± 0.226 (0.2 ~ 1.7)	0.24
CCT (microns)	536.5 ± 26.5 (485–617)	537.5 ± 24.2 (488 ~ 628)	0.76
Km(D)	43.5 ± 1.2 (39.1–47.15)	43.5 ± 1.4 (39.95 ~ 47.65)	0.98

T-test was used for normally distributed data, and chi-square test was used for sex

*SD* Standard deviation; *T-CAT* EX500 topography-guided ablation; *CWG* Amaris corneal-wavefront-guided ablation; *D* Diopter; *MRSE* Mean refractive spherical; *CDVA* Corrected distance visual acuity; *UDVA* Uncorrected distance visual acuity; *CCT* Central corneal thickness; *Km* Mean keratometry

^a^*P* value < 0.05 was considered statistically significant

### Corneal wavefront HOA

**Fig. 1:**
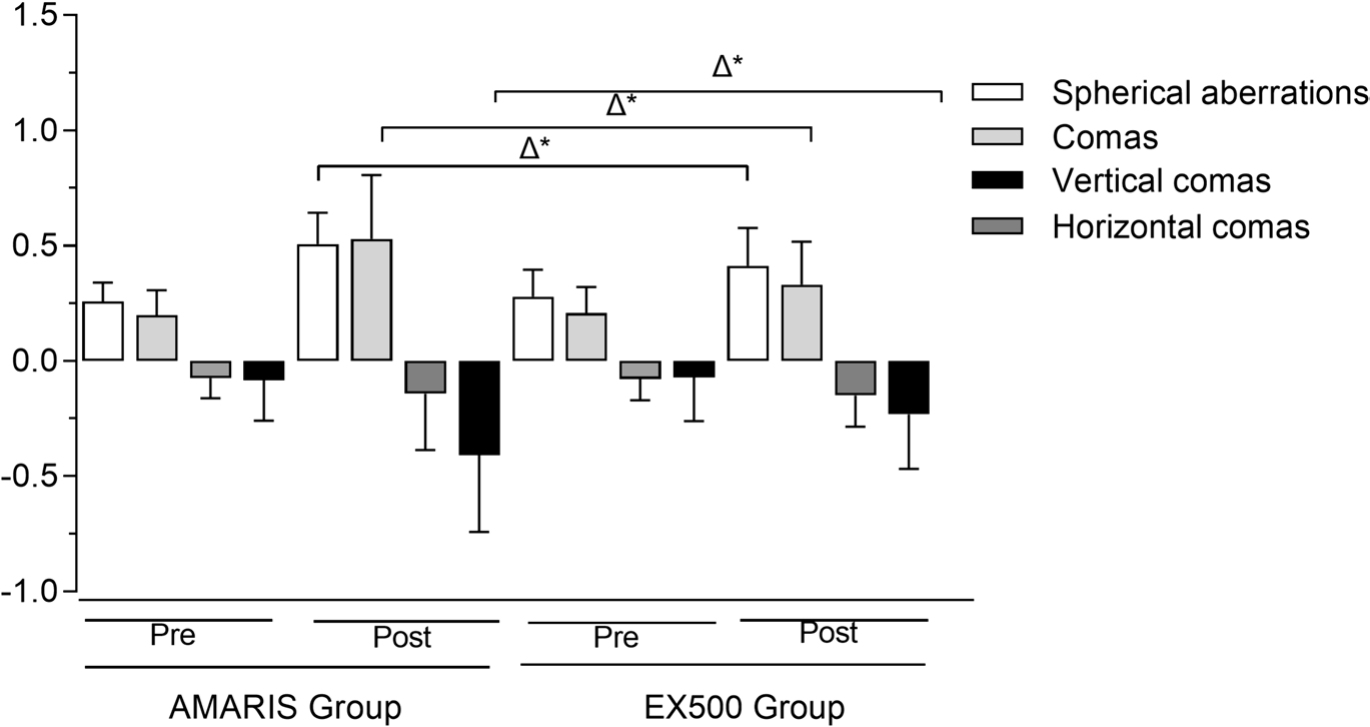
Corneal wavefront aberrations in AMARIS and EX500 groups. Δ^*^ means significant changes in aberrations before and after surgery, and the student t-test was used for the AMARIS and EX500 groups

Table 2 shows the preoperative and postoperative corneal HOAs in both the groups. After surgery, there were significant increases in spherical aberrations, coma aberrations, and vertical coma aberrations in both the WaveLight and AMARIS groups. Postoperatively, the coma aberrations (0.331 ± 0.186 vs 0.529 ± 0.278, T = 4.18, *P*<0.001) and spherical aberrations (0.412 ± 0.165 vs 0.507 ± 0.136, T = 0.15, *P* = 0.02) were significantly larger in the AMARIS group when compared with the WaveLight group. At 3 months postoperatively, the increase in spherical aberrations (0.104 ± 0.199 µm vs 0.254 ± 0.146 µm, T = 0.25, *P* < 0.05) and coma aberrations (0.117 ± 0.202 µm versus 0.316 0.297 µm, T = 0.70, *P* < 0.05) were significantly lower in the WaveLight group than that in the AMARIS group. Furthermore, the changing amplitude of postoperative spherical aberrations, coma aberrations, and vertical coma aberrations of the WaveLight group were lower than that of the AMARIS group (Fig. 1).

**Table 2:** Preoperative and postoperative corneal HOA in patients who underwent corneal wavefront/topography-guided LASIK, and were divided into the AMARIS and EX500 Groups respectively

AMARIS group					EX500 group			†P
Corneal aberration (um)	Preoperative	Postoperative	Post–Pre	*p*	Preoperative	Postoperative	*p*	
Coma	0.198 ± 0.108	0.529 ± 0.278	0.316 ± 0.297	< 0.001^*^	0.208 ± 0.113	0.331 ± 0.186	< 0.001^*^	< 0.001^*^
Spherical aberration	0.258 ± 0.081	0.507 ± 0.136	0.254 ± 0.146	< 0.001^*^	0.279 ± 0.116	0.412 ± 0.165	< 0.001^*^	0.02^*^
Horizontal coma (Z_3_^−1^)	− 0.074 ± 0.088	− 0.143 ± 0.243	0.051 ± 0.283	0.14	− 0.079 ± 0.091	− 0.149 ± 0.136	0.15	0.63
Vertical coma (Z_3_^1^)	− 0.083 ± 0.176	− 0.409 ± 0.332	0.330 ± 0.337	< 0.001^*^	− 0.071 ± 0.190	− 0.231 ± 0.238	< 0.001^*^	< 0.001^*^
|Horizontal coma (Z_3_^−1^)|	0.107 ± 0.076	0.233 ± 0.182	0.124 ± 0.328	0.17	0.111 ± 0.064	0.184 ± 0.128	0.36	0.09
|Vertical coma(Z_3_^1^)|	0.177 ± 0.117	0.444 ± 0.267	0.531 ± 0.457	< 0.001^*^	0.168 ± 0.113	0.243 ± 0.181	< 0.001^*^	< 0.001^*^

**Fig. 2:**
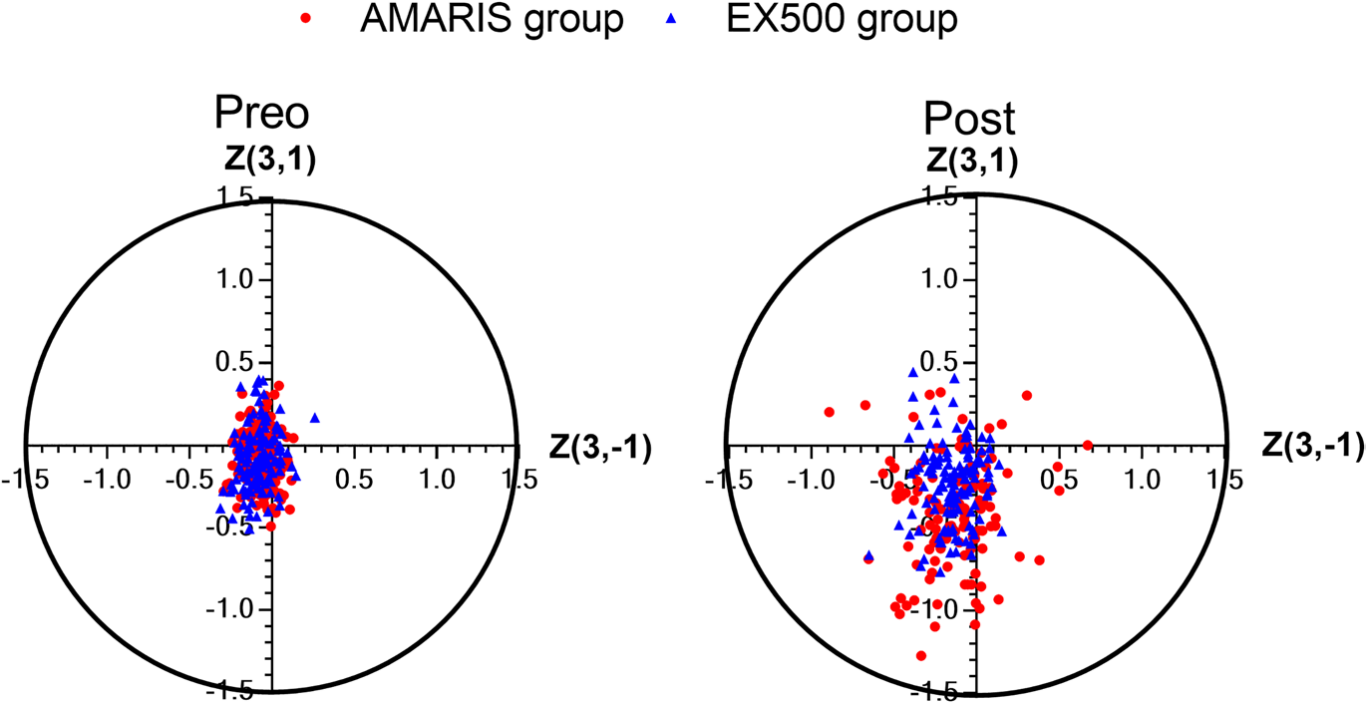
Horizontal and vertical coma distributions of the AMARIS and EX500 right eyes. Preo: preoperative; Post: postoperative; Z(3,−1)/Z(3,1): Zernike poly value of the horizontal coma/vertical coma of the central 6 mm corneal area assessed on a WaveLight Topolyzer Vario topographer

**Fig. 3:**
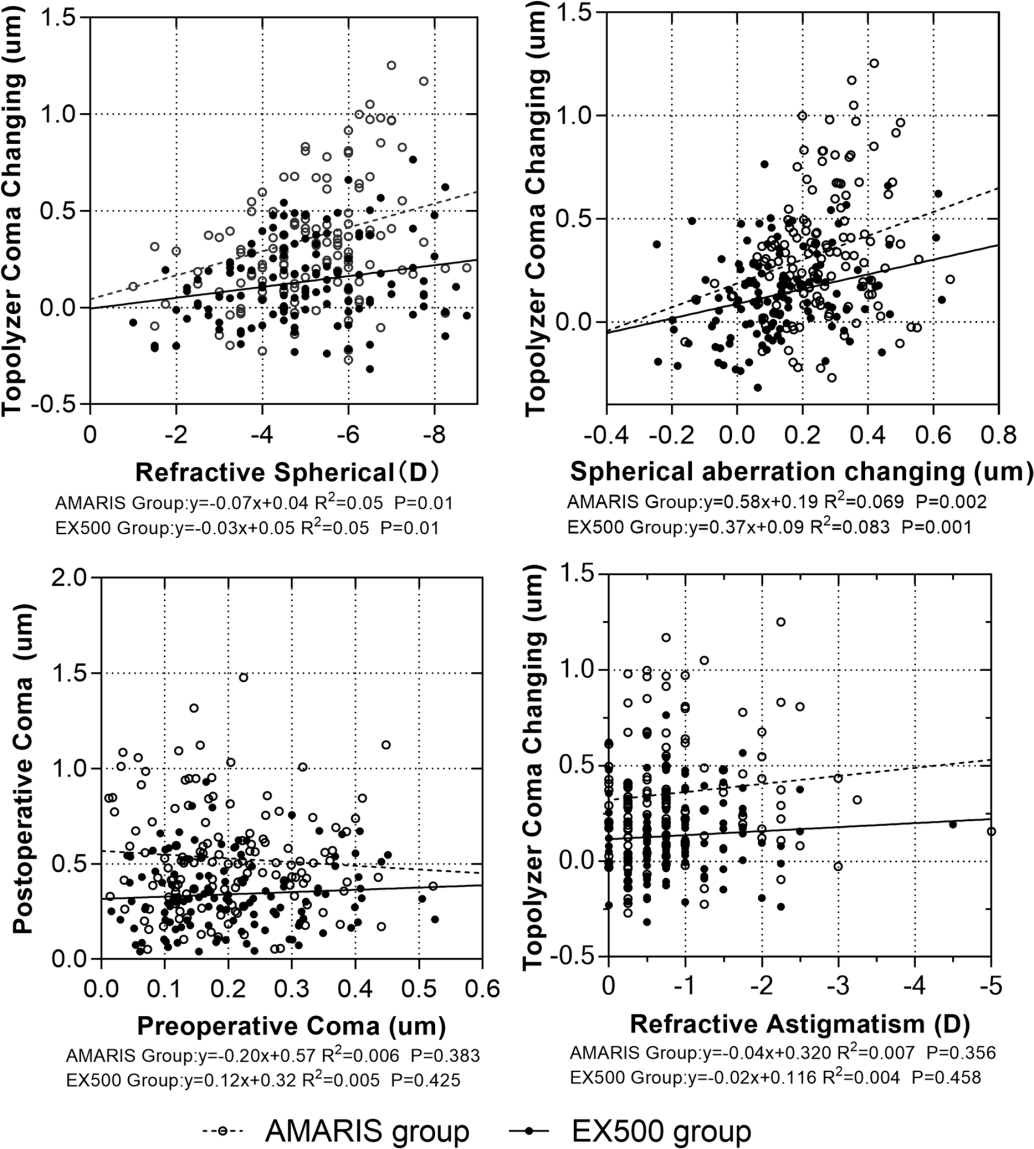
Linear regression analyses among comas, refractive spheres, and instances of astigmatism

Coma aberrations in the AMARIS group were offset and disperse (Fig. 2). In addition, a positive association between preoperative spherical aberrations and postoperative coma aberrations was observed in both the groups. The association revealed that for every 1 D change in preoperative spherical aberration, the postoperative coma aberration increased by 0.205 µm (y = 0.005 + 0.205x, *P* = 0.001, R^2^ = 0.05) and 0.415 µm (y = 0.044 + 0.415x, *P* = 0.001, R^2^ = 0.05) in the WaveLight and AMARIS group, respectively (Fig. 3). Furthermore, changes in the amplitude of the WaveLight group were smaller than those in the AMARIS group. Moreover, no association among the changes in coma aberrations, cylindrical, or preoperative coma aberrations in either group was observed (Fig. 3).

### Contrast sensitivity

**Fig. 4:**
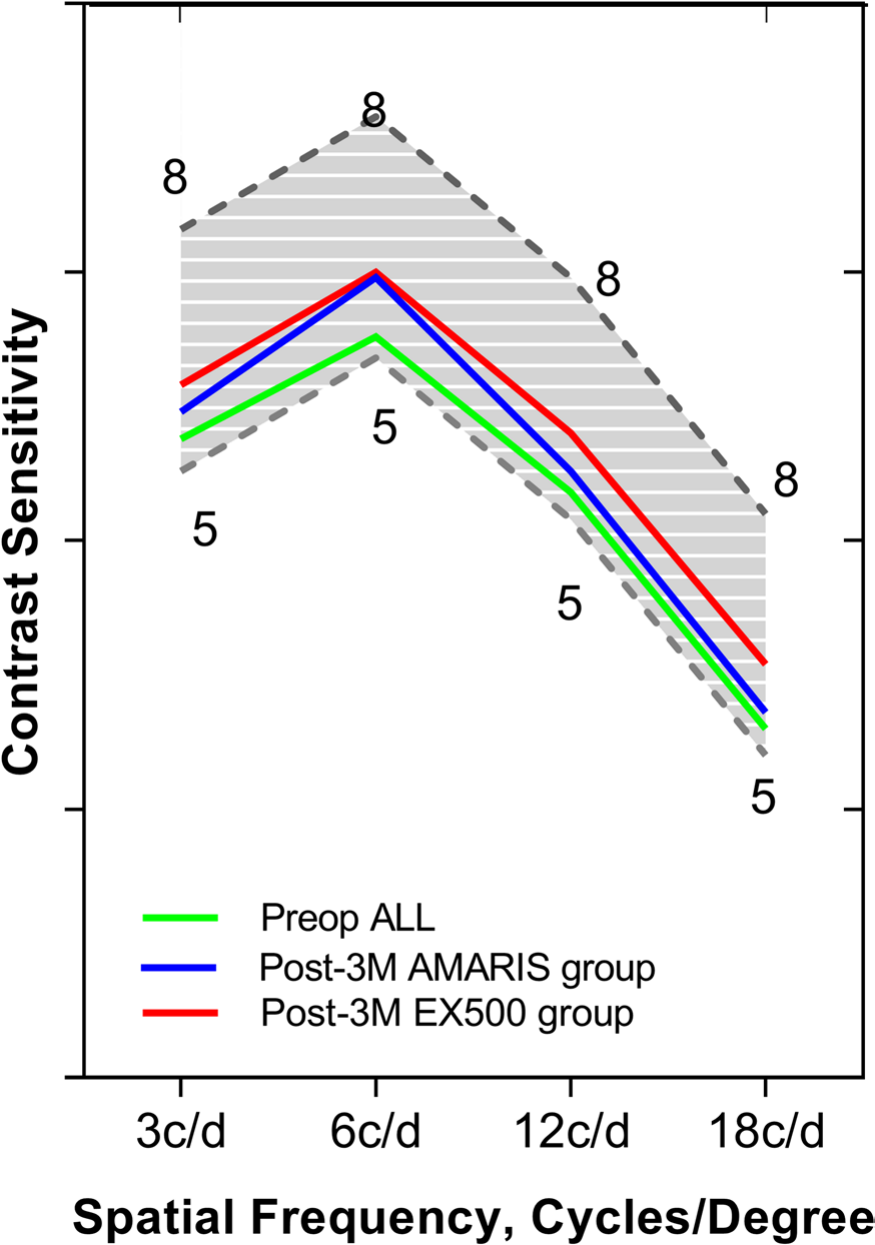
Topographic maps of EX500 and Amaris guided the contrast sensitivity results for different spatial frequencies before and after LASIK. Compared to preoperative levels, the contrast sensitivity of 3 c/d, 6 c/d, 12 c/d, and 18 c/d in the EX500 and AMARIS groups increased compared to those pre-operation (T =  − 2.55, − 3.61, − 3.79, − 3.96 in 3 c/d, 6 c/d, 12 c/d, and 18 c/d in contrast sensitivity of the AMARIS group, and T =  − 1.97, − 1.41, − 2.62 and − 1.86 in the EX500 group, and all *P* < 0.05); no differences of the contrast sensitivity among 3c/d,6 c/d, 12 c/d, and 18 c/d were observed (T = 4.23, 5.31, 5.45, 6.79 in 3 c/d, 6 c/d, 12 c/d, and 18 c/d between the EX500 and AMARIS groups, *P* ≥ 0.05); the student t-test was used to comparision between the AMARIS and EX500 groups

Figure 4 represents the contrast sensitivities under different spatial frequencies (3 c/d, 6 c/d, 12 c/d, and 18 c/d). The results obtained demonstrated that postoperative contrast sensitivities were better than preoperative contrast sensitivities in both the groups (Fig. 4).

### Visual acuity and refraction

**Fig. 5:**
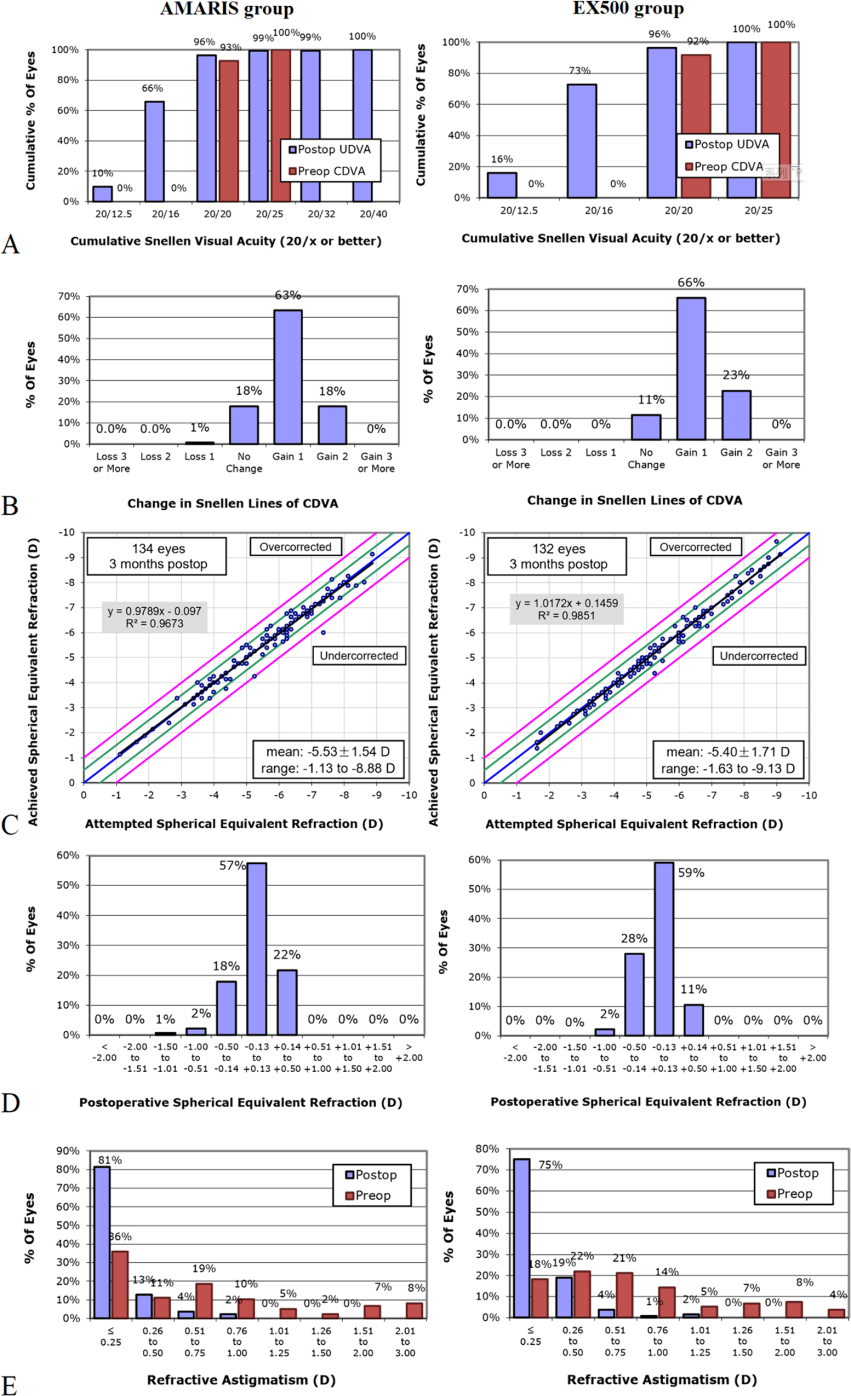
Outcomes of refractive surgery. **A** Uncorrected distance visual acuity. **B** Changes in the corrected distance visual acuity. **C** Attempted versus achieved spherical equivalent refraction. **D** Postoperative spherical equivalent refraction. **E** Refractive astigmatism

Figure 5 shows the refractive and visual outcomes in the WaveLight group and AMARIS group. At the end of the 3-month follow-up, both the procedures were equally efficacious, where, 127 of 132 (96.2%) eyes and 129 of 134 (96.3%) (X^2^ = 2.74, *P* = 0.51) eyes achieved a UDVA of 20/20 in EX500 and AMARIS groups, respectively. A total of 117/132 (88.6%) and 109/134 (81.3%) eyes gained 1 or more lines of postoperative UDVA compared with preoperative CDVA in the WaveLight and AMARIS group, respectively. The intergroup comparison with respect to the number of eyes gaining 1 or more lines of postoperative UDVA revealed a lack of statistically significant difference between the groups (88.6% vs 81.3%, X^2^ = 1.76, *P* = 0.70). At 3 months postoperatively, the mean postoperative MRSE was − 0.05 ± 0.21 and − 0.02 ± 0.28 (T = 17.57, *P* = 0.34) in the WaveLight group and AMARIS group, respectively. Moreover, the percentages of eyes having MRSE within ± 0.50 D measured at 3 months follow-up were 96.9% in EX500 group and 97.1% in the AMARIS group (X^2^ = 8.17, *P* = 0.89).

## Discussion

The different excimer laser platforms used for LASIK may have different characteristics with respect to the creation of the custom ablation profile, ablation strategies and other features specific to each platform. In this study, we compared the performance of WaveLight EX500 and Amaris 1050 s platforms with respect to the magnitude of postoperative HOAs and visual acuity after 3 months of surgery.

In this study, the increase in spherical and coma aberrations after 3 months of surgery were significantly lower in the WaveLight group when compared with the AMARIS group. A positive association was also observed between postoperative coma aberrations and preoperative spherical aberrations. This proposes that larger preoperative spherical aberrations may lead to increasing postoperative coma aberrations. These findings are in accordance with previous studies where a similar positive association between preoperative spherical aberrations and postoperative coma aberrations was observed [24, 25]. The possible reasons for the changes in postoperative aberrations were as follows: (1) There was a higher risk of aberration after LASIK, which is complex and multi-faceted (corneal flap and wound healing reactions can increase high-order wavefront aberrations); (2) changes in the postoperative corneal anterior surface may lead to a risk of increased postoperative spherical aberration and coma aberration [26, 27]; (3) due to different ablating modes and corneal aberration devices.

In this study, both the WaveLight Contoura EX500 and the Schwind Amaris 1050S LASIK were shown to provide excellent visual and refractive outcomes. After 3 months of follow-up, around 96% of eyes achieved 20/20 or better UDVA in both the groups and the postoperative MRSE was within ± 0.50 D in 96.9% and 97.1% of the eyes in WaveLight and AMARIS group, respectively. These results are comparable to previous studies which reported good visual acuity and refractive results after CTG surgeries [14, 15, 21]. Previously, an FDA study evaluated the visual and refractive outcomes of the ALCON Contoura T-CAT platform for the correction of astigmatism and myopia. The study reported that the percentage of eyes achieving a mean UDVA of 20/20 or better was more than 93%, and more than 94% of patients had a postoperative MRSE within ± 0.50 D at 3 months after the surgery [14, 15]. Similarly, Zohu et.al. reported that 95% of eyes achieved UDVA of 20/20 or better and the postoperative MRSE was within ± 0.50 D in 95% of the eyes after using Schwind Amaris CWG platform in patients with myopia and myopic astigmatism [21]. In the current study, the outcomes with respect to the percentage of eyes achieving an UDVA of 20/20 with a postoperative MRSE within ± 0.50 D may have been relatively lower due to the inclusion of patients with a minimum preoperative CDVA of 20/25.

Previously, Blanton et al. had conducted a meta-analysis that compares 4 different excimer laser platforms and revealed that the visual acuity of WaveLight was better than the AMARIS, which was in accordance to the current study [22]. We suggest that the differences in visual acuity may have been due to the larger HOA of the Amaris 1050S group compared with the WaveLight group, as HOAs reduce visual quality [23].

In the present study, WaveLight EX500 was used in the symmetric offset mode (SO) which centered on the corneal vertex with a regular ablating circle area. The Amaris platform was used in asymmetric offset mode (AO), which was an asymmetrical ablating circle that centered on the corneal vertex, cutting across the pupil boundary. Moreover, results from the current study indicated that when selecting AO, increased coma aberration and trefoil may occur due to aspheric defocus. Our results are in agreement with previous studies wherein the rates of horizontal coma aberration and vertical trefoil were reported to increase postoperatively in a corneal aberration-guided LASIK correction for hyperopia and astigmatism using the AO mode by AMARIS [20, 28]. More recently, we reported that the changing amplitudes of postoperative coma aberration and trefoils of the Schwind Amaris 1050 s platform with the asymmetric centration were larger than those with the symmetric centration with Schwind Amaris 1050 s and WaveLight EX500 [19]. Previous studies have shown that the vertical offset of both the AMARIS and WaveLight excimer platforms (the apex of the cornea offset from the center of the entrance pupil plane) were greater than the horizontal offset, while vertical offset compensations were less than the horizontal compensation. These could cause vertical ablation offsets and corneal irregularities in the vertical direction, resulting in changes in the vertical coma aberration [2, 29].

The major strengths of our study are-(1) its larger sample size and (2) the visual outcomes associated with both platforms come alongside with graphical and tabular representation of data in an unbiased format. (3) The personalized module design guided by topographic maps of different excimer platforms and the subtle differences in actual results can be provided to refractive surgeons, so as to select different excimer platforms in the face of different patients and provide more reference for surgical design, so as to obtain better surgical results. However, our study has certain limitations which include a short-term follow-up and the non-comparable nature of different aberration devices. In previous studies, we can also found that with the period of followed-up, the results including the refraction and corneal HOAs have changes. In Sharif et al.’s study ^31^, it was found that postoperative myopic regression increased over time, and the mean MRSE was -0.36 D at 6 months after surgery compared -0.34 D at 3 months after surgery, which may be related to corneal epithelial remodeling, corneal biomechanics, etc. In order to better evaluate the difference between the two excimer platform, we need more long-term results in our future research. Different excimer platforms were theoretically more compatible with their respective corneal topographer and aberration devices. The use of Vario Topolyzer, which was a compatible match to the WaveLight EX500 platform, in testing pre- and post-operative aberration of WaveLight and Amaris platforms may have probably limited the optimum function AMARIS platform. We would also like to highlight that there is no perfect aberration device and hence the surgical outcomes of different platforms may be accurately determined only if different aberration devices were employed in the same study.

## Conclusions

To conclude, EX500 CTG LASIK and Amaris CWG LASIK achieved excellent vision, refraction, and contrast sensitivity. Lastly, in regards to HOAs, the EX500 showed minimal spherical aberrations and a low coma occurrence.
